# Rollator usage lets young individuals switch movement strategies in sit-to-stand and stand-to-sit tasks

**DOI:** 10.1038/s41598-023-43401-6

**Published:** 2023-10-06

**Authors:** Michael Herzog, Frieder C. Krafft, Bernd J. Stetter, Andrea d’Avella, Lizeth H. Sloot, Thorsten Stein

**Affiliations:** 1 BioMotion Center, Institute of Sports and Sports Science, Karlsruhe Institute of Technology (KIT), Engler-Bunte Ring 15, 76131 Karlsruhe, Germany; 2 HEiKA-Heidelberg Karlsruhe Strategic Partnership, Heidelberg University, Karlsruhe Institute of Technology (KIT), Karlsruhe, Germany; 3 Optimization, Robotics, and Biomechanics, Institute of Computer Engineering, Heidelberg University, Heidelberg, Germany; 4 Center of Prevention, Diagnostic and Performance, Center of Orthopaedics Hohenlohe, Künzelsau, Germany; 5 Sports Orthopedics, Institute of Sports and Sports Science, Karlsruhe Institute of Technology (KIT), Karlsruhe, Germany; 6 Laboratory of Neuromotor Physiology, IRCCS Fondazione Santa Lucia, Rome, Italy; 7 Department of Biomedical and Dental Sciences and Morphofunctional Imaging, Università di Messina, Messina, Italy

**Keywords:** Geriatrics, Quality of life, Therapeutics, Biomedical engineering, Computational neuroscience, Learning and memory, Motor control, Sensorimotor processing, Somatosensory system

## Abstract

The transitions between sitting and standing have a high physical and coordination demand, frequently causing falls in older individuals. Rollators, or four-wheeled walkers, are often prescribed to reduce lower-limb load and to improve balance but have been found a fall risk. This study investigated how rollator support affects sit-to-stand and stand-to-sit movements. Twenty young participants stood up and sat down under three handle support conditions (unassisted, light touch, and full support). As increasing task demands may affect coordination, a challenging floor condition (balance pads) was included. Full-body kinematics and ground reaction forces were recorded, reduced in dimensionality by principal component analyses, and clustered by k-means into movement strategies. Rollator support caused the participants to switch strategies, especially when their balance was challenged, but did not lead to support-specific strategies, i.e., clusters that only comprise light touch or full support trials. Three strategies for sit-to-stand were found: forward leaning, hybrid, and vertical rise; two in the challenging condition (exaggerated forward and forward leaning). For stand-to-sit, three strategies were found: backward lowering, hybrid, and vertical lowering; two in the challenging condition (exaggerated forward and forward leaning). Hence, young individuals adjust their strategy selection to different conditions. Future studies may apply this methodology to older individuals to recommend safe strategies and ultimately reduce falls.

## Introduction

Falls are the leading cause of unintentional injuries in older individuals, often causing hospitalization and death^1–3^. Approximately 30% of individuals over 65 years old fall at least once a year^4,5^.

Risk factors include lower-body weakness and impaired balance^3,6,7^. Rollators, or four-wheeled walkers, are often prescribed for patients needing gait assistance^8^ to reduce lower-limb loading, compensate for weakness and injury, and improve balance^8–10^. They aim to empower the residual motor capacities and facilitate natural locomotion^11^. Rollator users can shift the load from the lower body to the upper body^12^ and increase their base of support by using the handles as additional contact points^8^. However, rollators paradoxically have also been found to be a risk factor for falls^6,8^. It is not yet clear what underlies the increased fall risk and how rollator usage affects movement coordination during different tasks^13^, as evaluations of rollator usage have been mostly limited to spatiotemporal parameters during walking^13^. Furthermore, a recent review points to a lack of data on gait aid prescription relative to fall risk or balance performance in older individuals^14^.

To start walking, we often need to stand up from a sitting position and sit down afterward. Hence, standing up and sitting down are fundamental tasks in daily life essential for mobility. Indeed, non-disabled adults stand up 60–100 times a day^15,16^. Devices like sit-to-stand lifts, rails, and exoskeletons are available to provide specific assistance with standing up^17^. But these devices have disadvantages, such as being expensive and needing installation, space, batteries, and power outlets^17^. Thus, in many situations, individuals may want or need to use a rollator to stand up and sit down. However, the transitions between standing and sitting are complex. Difficulties standing up correlate with the risk of falling^18^ and increase the need for assistance^19^, leading to reduced independence and earlier institutionalization^20^. Studies in residential aged care facilities report that 21–41% of falls happen during transfers^21–23^. Lehtola et al.^24^ reported the second-highest fall risk for sit-to-stand and stand-to-sit transfers in home-dwelling adults over 85 years.

Transitioning between sitting and standing becomes more demanding with increasing age, mostly due to changes in muscle composition and a decline in motor control^25,26^. Knee and hip extension muscles provide less force to stand up^27^, and knee extensor activity correlates with stability in sitting down^25^. Transitions between sitting and standing require the simultaneous, coordinated motion of the lower extremities and the upper body^28,29^. Standing up means shifting the center of mass (CoM) from above the buttocks to above the feet by hip flexion and anterior movement of the head-arms-trunk segment, followed by rising through the extension of the hips, knees, and ankles^28–32^. This poses a challenge to balancing as the body transfers from a stable seated position with three contact points (feet and buttocks) through an unstable, dynamic movement with the CoM outside the base of support to standing, where the base of support is smaller, the CoM is higher, and only two contact points are used^15,33,34^. Sitting down requires a balanced and smooth transition of the CoM from above the feet onto the chair seat. However, sitting down cannot be assumed to be simply the opposite of standing up^35^. Sitting down is performed with—rather than against—gravity, and the transition phase begins with a trunk flexion while standing, which is a less stable position than sitting^35^. Thus, three questions arise on how these complex movements are executed and if rollator use can improve the transition between sitting and standing.

First, for standing up, individuals have been observed to use different strategies: (1) leaning or sliding forward, (2) upper body flexion, (3) moving the feet backward, and (4) pushing through the arms on armrests^33,36–38^. When the feet remain parallel and unchanged throughout standing up, two main strategies were observed: (1) the forward leaning, or momentum transfer strategy, and (2) the (dominant) vertical rise strategy, which has also been called the force control strategy^33,37,39,40^. With the forward leaning strategy, individuals use the upper body to generate forward momentum, which is smoothly transferred to mostly vertical momentum after seat-off by extending the knees and hips^28,39,41^. Thereby, as the thorax, abdomen, and pelvis rotate in the same direction during the forward lean, energy is transferred from the thorax to the thigh (mechanical energy flow^39,42^). As a result, knee torque is reduced^43^ compared to a rise solely by lower-body muscles (vertical rise strategy). In the latter, the trunk stays relatively vertical throughout the movement^33,44^, and the CoM shows only anterior and upward movement^39^ but no downward movement before it rises. The forward leaning strategy has generally been said to be the most efficient strategy for healthy individuals, as demonstrated in various studies^43^. It is especially efficient, such that it reduces the knee torque compared to rising without the help of upper body momentum^39,43^. Enough muscle strength in the knee extensors is one of the most dominant factors for a successful transfer^45^. A rollator may help individuals use the advantageous forward leaning strategy, with less force required, and still perform the movement safely.

Secondly, as described earlier, sitting down is also a complex movement and cannot be assumed to be simply the opposite of standing up^35^. It is, therefore, even more surprising that sitting down has been little described in the biomechanical literature^35,37^, let alone when using a rollator. Possibly, there are different strategies for sitting down, which may also be influenced by rollator use.

Thirdly, observational studies have often described arm use in transitioning between sitting and standing^46^. However, arm movement is rarely described in biomechanical analyses since these are mostly restricted for standardization purposes^37,40^. Yet, arm swing or push might play a vital role in easing the generation of horizontal and vertical momentum^47^. Indeed, Wretenberg et al.^48^ found a 44% and 34% reduction in hip and knee load, respectively, when their participants could push on armrests. Aging muscles often weaken^49,50^, meaning that 50% of healthy older individuals cannot stand up without using their arms if the seat is at knee height^51^. This makes armrests a necessity. Hence, arms play a major role in generating propulsive forces during standing up, which can compensate for deficits in lower body strength^48,52,53^. Also, the additional contact point of the hands helps maintain balance^52,54^ through somatosensory input by a tactile or proprioceptive haptic cue^8,55,56^. In walking and standing, it has been shown that touching a plate or railing with the fingertip improves balance rather than offering mechanical support when allowing strong interaction forces^57,58^. In support, van der Kruk et al.^59^ recently observed that the preference of arm use is likely related to the perception of stability and the fear of falling. Thus, rollator handles could greatly simplify standing up and sitting down via mechanical and/or proprioceptive support. However, the few studies that include arms in a biomechanical analysis used armrests rather than rollator handles. Armrests are low and directly lateral to the trunk when sitting, in contrast to anterior-lateral and high as with a rollator^48,54,60^. Our previous study^61^ found that movement stability (i.e., CoP_feet_-length, duration) was enhanced by full rollator support in standing up and sitting down. However, the strategies individuals use when arm support is given are not yet known. Potentially, there exist strategies which make transitions manageable and safe.

This study’s purpose was to investigate how rollator support affects strategies for sit-to-stand and stand-to-sit movements. We aimed to identify the strategies used when the rollator usage level changes from no assistance to light touching (supposed proprioceptive cue) and full support (supposed load reduction). Due to the lack of biomechanical studies on movement strategies during standing up and sitting down and the heterogeneity in the physical status of older individuals, this first study investigated non-disabled young adults. As older individuals often struggle with balance, we added a balancing aspect to make the transitions challenging. To mimic age-related balance problems, icy water or convex lenses have been used^62^, and for aging suits, a foam material is glued to the bottom of shoes to create imbalance^63^. There is agreement that proprioception signals from leg muscles provide an essential source of information for postural control^64,65^. Hence, we aimed to trigger the proprioceptive component, focusing more generally on postural instability, which can result in falls and injuries^64^. Therefore, we used balance pads in addition to the normal floor condition.

We hypothesized that (1) individuals switch their strategy depending on the level of rollator assistance, (2) we would find specific strategies for (2a) the different degrees of rollator assistance, and (2b) floor condition.

## Materials and methods

We used the same raw and pre-processed data described in our previously-published article^61^. All steps after pre-processing (“Data processing”) are novel in this article.

### Participants

**Figure 1:**
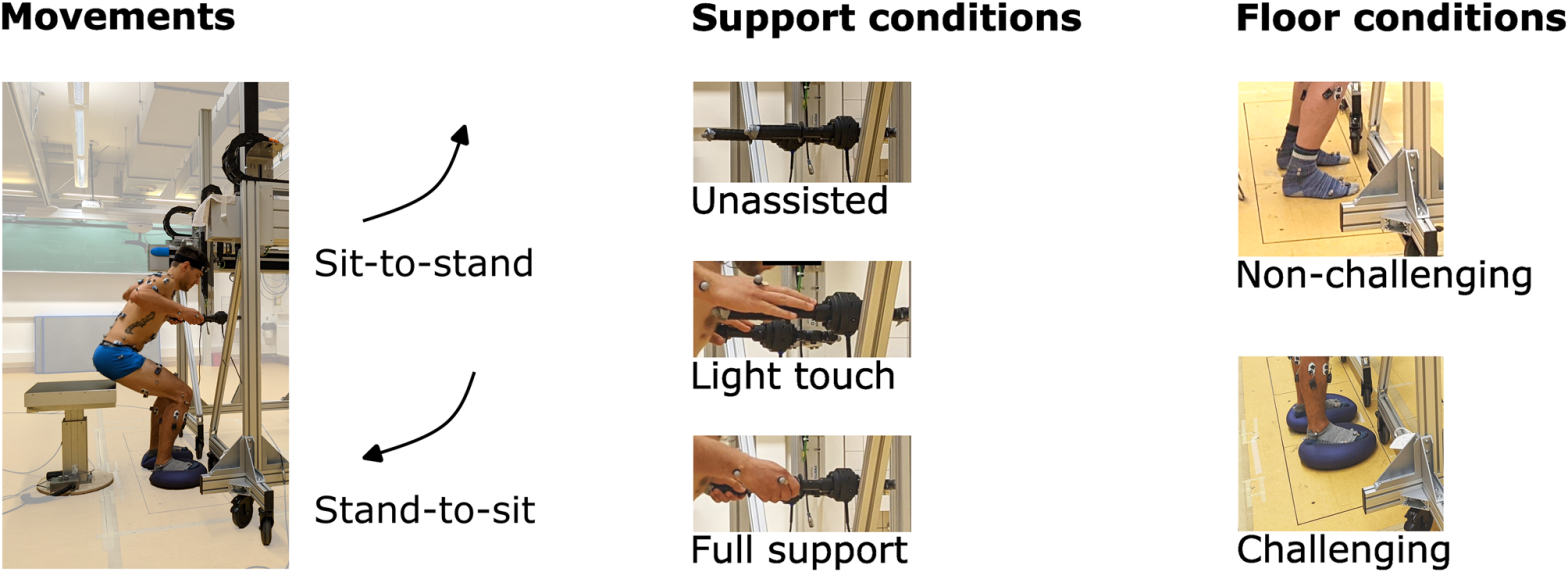
Experimental setup. Left: Participant stands up from an instrumented chair with the custom-made robot rollator simulator. Full-body passive markers for motion tracking and EMG electrodes (data not included in this article) were placed on the body. Two movements were studied: sit-to-stand and stand-to-sit. Middle column: Three different support conditions were used: unassisted (top, handles not used), light touch (middle, palm on the handles), and full support (bottom, power grip). Right column: Two floor conditions were used: non-challenging (lab floor, top) and challenging (balance pads, bottom).

Ten females and ten males (25.5 ± 3.8 years, 1.71 ± 0.08 m height, 67.6 ± 10.9 kg mass) participated in the study and gave written informed consent before participation. The number of participants was selected based on comparable, recent biomechanical studies^25,66,67^. Informed consent was obtained by the participant shown in Fig. 1 to publish the image. The study was approved by the Ethics Committee of the Medical Department of Heidelberg University (S-105/2021) and has been performed in accordance with relevant guidelines/regulations and the Declaration of Helsinki.

### Experimental protocol

The participants stood up and sat down at their preferred speed, separated by at least two seconds of rest as instructed with the commands “stand up, stand still, sit down.” They used a custom-made robot rollator simulator under three different support conditions (Fig. 1). The participants were instructed not to use the rollator in the (1) unassisted condition and to let their arms hang loosely at their sides as long as they sat. In the (2) light touch condition, they were instructed to place their hands with a palm grip onto the handles to induce a proprioceptive cue, and in the (3) full support condition, they were instructed to use a power grip on the handles. Handle height was individually set at the participant’s wrist height in a standing position as recommended by health care literature^68–70^. Seat height was individually adjusted to the height of the lateral epicondyle of the femur. To induce a higher demand on balance capabilities, an additional “challenging condition” was set up by placing a circular rubber-made balance pad with a compliant surface (Dynair^®^ Ballkissen^®^, diameter 33 cm, height 8 cm, TOGU GmbH, Prien-Bachham, Germany) underneath each foot. Participants performed two trials in each condition to familiarize themselves with the setup. Everyone performed three valid, non-consecutive trials in a random order for every support (unassisted, light touch, full support) and floor condition (non-challenging and challenging).

### Data collection

Full-body 3D kinematics were recorded (150 Hz, 10 type 5 + cameras, Qualisys, Gothenburg, Sweden) using retroreflective markers placed according to the IOR full-body gait model^71,72^. Ground reaction forces (GRF) (1000 Hz; Bertec Corp., Columbus, OH, USA) and forces on the seating surface (142 Hz; Phidgets Inc., Calgary, AB, Canada) were measured.

### Data processing

Raw kinematic data were processed offline with Qualisys Track Manager (v2018.1, Qualisys Medical Ltd., Sweden) to reconstruct the 3D coordinates of the markers. Subsequently, force and kinematic data were filtered with a 4th-order, 10 Hz, Butterworth low-pass filter. Full-body kinematics and the CoM were then calculated with Visual3D (v6, C-Motion Inc., Germantown, MD, USA). Further data analyses were done in Matlab (R2020a, Natick, MA, USA). Movement start, seat-off, and movement end were segmented using a k-means++ algorithm^73^. The trials were time-normalized to 1001 time points (100%).

### Data analysis

The following variables were selected for further analyses as they have been described as determinants for the strategies: 3D ground reaction force, normalized to body weight^74,75^, the 3D CoM, normalized to body height^31,33,34,76^, sagittal angles of the ankle, knee, and hip^40,44,77^, the sagittal angle between a virtual line connecting the heel and the CoM and the floor perpendicular (“CoM-heel angle”)^39,78^, and the sagittal angles of thigh, shank, and pelvis relative to the floor perpendicular^38^. We focused on the right body side as their values were similar to those of the left side. Kinematic time series of arm movements were not included in the analysis to identify movement strategies. They would have provoked a strict separation between unassisted and light touch/full support trials and thus obscured the similarities of the trials across all support conditions regarding whole-body strategies.

#### Identification of movement strategies

**Figure 2:**
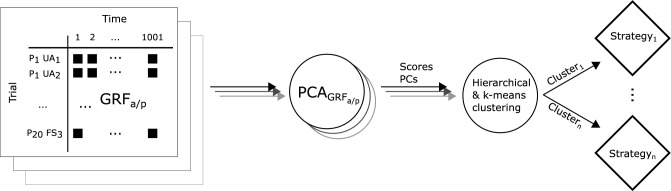
Process for identifying strategies, performed separately for each of the four combinations of sit-to-stand, stand-to-sit, and non-challenging, challenging floor conditions. For each variable, a matrix (e.g., GRF_a/p_) served as input for the variable-specific PCA (PCA_GRFa/p_). The input for the clustering consists of a coordinate system with axes formed by the extracted principal components of all variables, as well as the scores obtained from the PCAs. The extracted clusters constitute the strategies. *P*_*i*_ ith participant, *UA*_*i*_ ith unassisted trial, *FS*_*i*_ ith full support trial.

Principal component analysis (PCA) was used to reduce the dimensionality of the data by extracting relevant features. These features were then used to identify movement strategies through k-means++ clustering (Fig. 2)^79–81^. This was done separately for the two movements (sit-to-stand and stand-to-sit) and floor conditions (non-challenging and challenging).

First, the data of each variable was arranged into a matrix ℝ^180×1001^, in which every column represented one timepoint (of 1001 timepoints) and every row one trial (20 participants × three support conditions × three repetitions = 180^79,80^). After subtracting the mean of the matrix^82,83^, PCA transformed the data into a low-dimensional coordinate system spanned by principal components^84–86^. We selected the number of principal components so that 90% of the variance was explained (see Table 1 for an overview of the number of principal components^85,86^). Following previous studies^80,84,87^, a single coordinate system was spanned from the PCs of each variable. The scores, i.e., the original data represented in the new coordinate system, were scaled to the interval [0,1] so that each extracted PC had equal importance in the subsequent clustering^88^. First, hierarchical clustering (ward distance) was used to determine the appropriate number of clusters based on the scree and silhouette coefficient plots. Then, k-means++ (Matlab kmeans with the ‘plus’ option) clustering grouped all 180 trials into the determined number of clusters. Each cluster represented one movement strategy. If the strategy of a single trial differed from all identified strategies, it was still assigned to one of the strategies because k-means does not terminate until every trial is assigned to a cluster. These outlier strategies were identified after clustering with the generalized extreme Studentized deviate test for outliers based on their distance to the centroid^89–91^. Clustering was repeated ten times to confirm the robustness of the cluster assignments^80^.

**Table 1:** Number of principal components necessary to explain 90% of the variance within the time series of every selected variable.

	Sit-to-stand		Stand-to-sit	
	Non-challenging	Challenging	Non-challenging	Challenging
GRF medio-lateral	3	5	5	8
GRF anterior–posterior	4	6	6	8
GRF vertical	3	4	4	5
CoM medio-lateral	2	2	2	2
CoM anterior–posterior	2	2	2	2
CoM vertical	2	2	2	3
Ankle angle sagittal	3	3	3	3
Hip angle sagittal	2	2	2	3
Knee angle sagittal	2	2	2	3
Pelvis-lab angle sagittal	2	2	3	3
Shank-lab angle sagittal	2	2	2	3
Thigh-lab angle sagittal	2	3	2	3
CoM-heel angle sagittal	2	2	2	2

#### Dependent variables

This study aimed to identify if participants switched strategy with support condition. A participant was identified as switching strategies if at least two of the three repetitions of one support condition had a different strategy than in another support condition.

Further, total trial duration and the duration between start and seat-on or -off in seconds were assessed because prolonged times have been associated with increased fall risk and less movement stability^61,92–94^. As arm movement in the unassisted condition was not restricted to allow for a “natural” movement, it was operationalized as a dichotomous variable: Arms were identified as “involved” when the most anterior elbow position was anterior to the shoulder.

### Statistics

Independent tests were used to assess differences between the identified strategies in the time series and dependent variables because trials, not participants, were clustered into strategies (see “Most of the participants switched their movement strategies”). First, analyses of the results found that the distributions of the individuals’ trials are different across the participants (see “Most of the participants switched their movement strategies”). Hence, dependent tests cannot be used. Therefore, independent tests were used that do not eliminate individuals as a source of variability, which results in less statistical power and thus are more conservative regarding significance than dependent tests. Normality was assessed with Shapiro–Wilk tests, and the homogeneity of variances was confirmed with Levene’s tests. Differences between the time series of the respective strategies were assessed with the spm1d toolbox (statistical parametric mapping, SPM^95,96^). As normality was violated, the non-parametric SPM versions of the ANOVA (three strategies) and t-tests (two strategies) were used^97,98^. Post-hoc pairwise comparisons by SPM were only performed on regions of interest indicated by the SPM–ANOVA^99,100^. In line with recent recommendations^101^, the aim of applying SPM was to exploratively analyze where the time series tended to differ, to help with the qualitative description of the strategies. Differences in the dependent variables, i.e., trial durations and time until seat contact, were tested with the Kruskal–Wallis and Mann–Whitney U–tests as homogeneity was violated. The significance level (two-tailed) was set a priori at 0.05 and adjusted for multiple comparisons post-hoc to 0.05/3 = 0.017 (Bonferroni correction). All statistics were performed in Matlab (R2020a, Natick, MA, USA).

## Results

Overall, in sit-to-stand, three strategies in the non-challenging and two in the challenging condition were identified (non-challenging: silhouette coefficient = 0.23, scree test SSE = 0.01; challenging: silhouette coefficient = 0.19, scree test SSE = 0.33). In stand-to-sit, three strategies in the non-challenging and two strategies in the challenging condition were also identified (non-challenging: silhouette coefficient = 0.18, scree test SSE = 0.31; challenging: silhouette coefficient = 0.19, scree test SSE = 1.30). The assignment of trials to clusters was robust throughout each instance of the 10 clustering runs.

### Most of the participants switched their movement strategies

**Figure 3:**
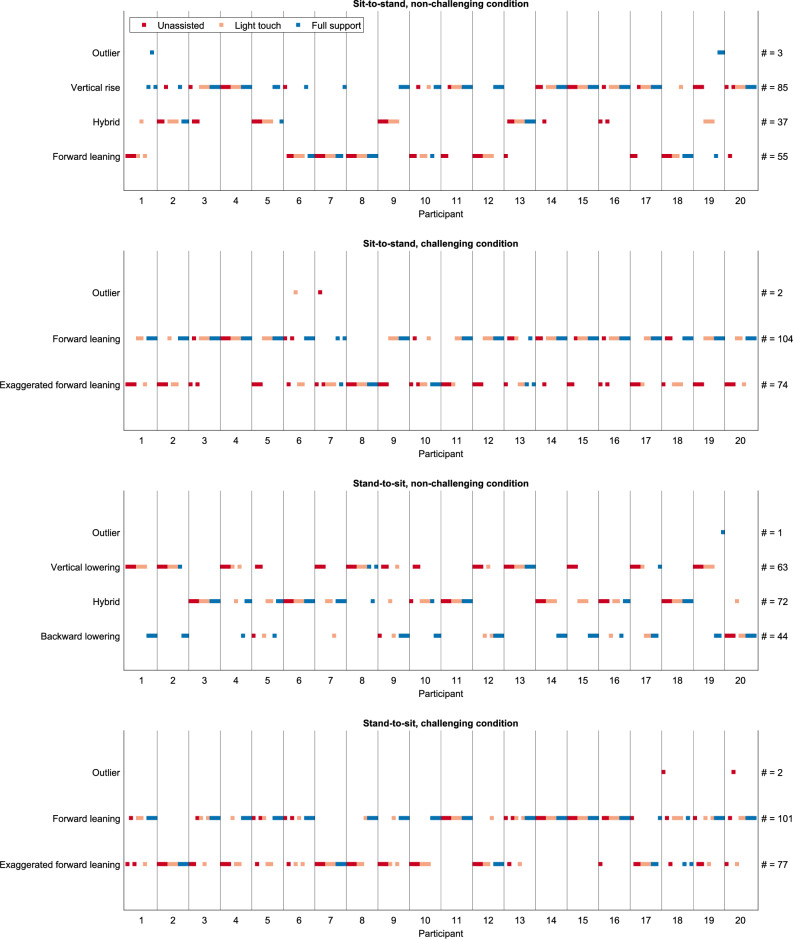
Distribution of trials among strategies. One dot represents one trial. The row indicates the strategy to which it belongs. The column shows to which participant it belongs. The support conditions are color coded as indicated by the legend. The labels on the right y-axis show how many trials were associated with the strategy written on the left y-axis.

Figure 3 illustrates which strategy was used for every trial. Of the 20 participants, between 14 and 17 (depending on the task) did not use just a single strategy for all their trials. Therefore, we accept our first hypothesis that individuals switch their strategy depending on the level of rollator assistance.

### Description and comparison of the identified movement strategies

#### Sit-to-stand movement strategies

**Figure 4:**
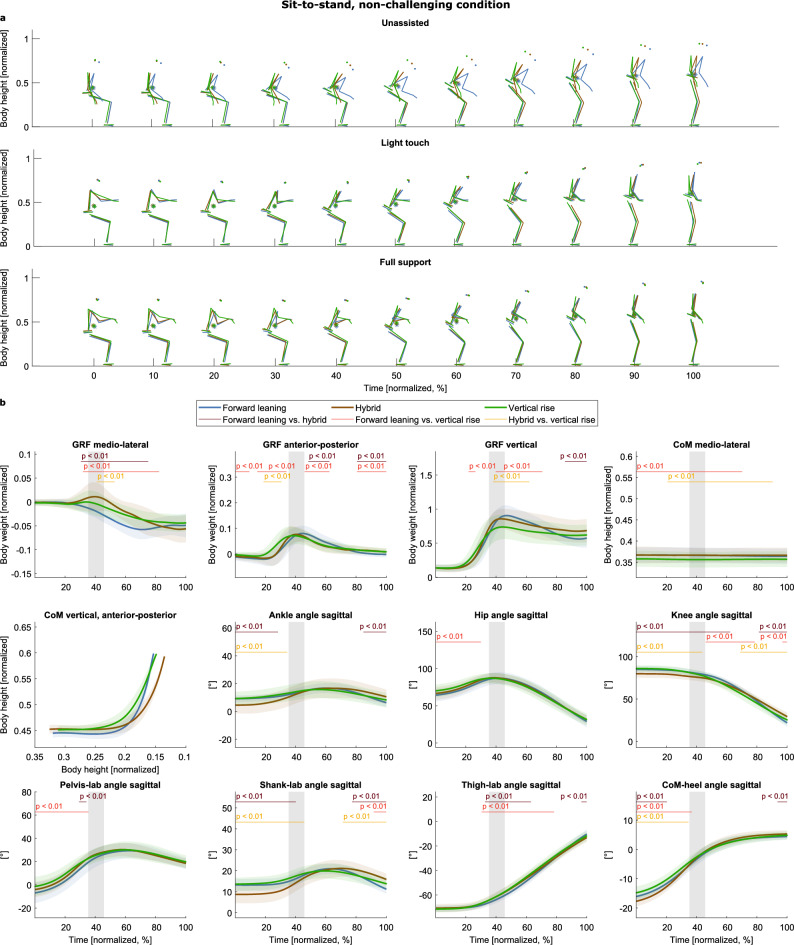
Strategies of the non-challenging sit-to-stand task. (**a**) The strategies are given in different colors (see legend) and separated into rows according to the support condition. The CoM is depicted as an asterisk (*). (**b**) Means and standard deviations of the variables, aggregated by cluster. The gray shaded area illustrates the range of seat-off (mean ± s.d.). The red lines and corresponding p-values indicate significant differences revealed by the post-hoc tests (p < 0.017).

Three different strategies were identified for sit-to-stand in the non-challenging condition (Fig. 4a, Supplementary Video “Sit-to-stand_non-challenging_condition.gif”): a “forward leaning” strategy (blue), a “vertical rise” (green) strategy, and a “hybrid” (brown) strategy, inferred by visual inspection of the stick figures and supported by the different courses in the hip and CoM-heel angles before seat-off and the CoM trajectory in the sagittal plane (Fig. 4b). The kinematics and stick figures of the “hybrid” strategy are either in-between the other two strategies or change from being closer to the one to being closer to the other over the course of the trial, thus named the “hybrid” strategy. These strategies appeared in each support condition, i.e., no strategy was identified to comprise only trials with handle support.

The trial duration with the forward leaning strategy was shorter than the other strategies. However, seat-off timing was not different between strategies (trial duration: forward leaning: 1.16 s ± 0.25, hybrid: 1.24 s ± 0.20, vertical rise: 1.24 s ± 0.22; χ^2^(2) = 10.37, p = 0.006; forward leaning vs. hybrid: z = −2.40, p = 0.016; forward leaning vs. vertical rise: z = − 3.04, p = 0.002; hybrid vs. vertical rise: z = 0.04, p = 0.969; seat-off: forward leaning: 0.49 s ± 0.13, hybrid: 0.50 s ± 0.11, vertical rise: 0.50 s ± 0.12; χ^2^(3) = 0.23, p = 0.892).

**Figure 5:**
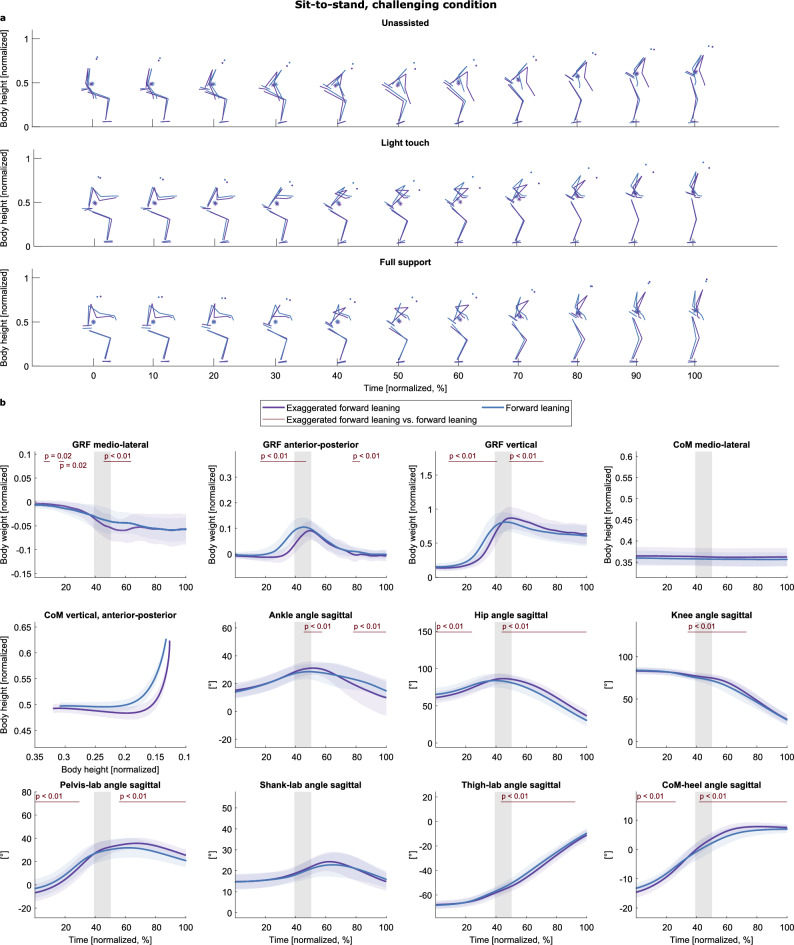
Strategies of the challenging sit-to-stand task. (**a**) The strategies are given in different colors (see legend) and separated into rows according to the support condition. The CoM is depicted as an asterisk (*). (**b**) Means and standard deviations of the variables, aggregated by cluster. The gray shaded area illustrates the range of range of seat-off (mean ± s.d.). The red lines and corresponding p-values indicate significant differences (p < 0.05).

In the challenging condition, only two strategies were identified (Fig. 5a, Supplementary Video “Sit-to-stand challenging_condition.gif”): an “exaggerated forward leaning” (purple) and a “forward leaning” strategy (blue). In comparison, the exaggerated forward leaning strategy shows a smaller hip angle in the transition phase after seat-off. The wide forward lean is underpinned by the CoM progression in the sagittal plane and the larger CoM-heel angle after seat-off Fig. 5b). Similar to the normal floor condition, no support-specific strategy was identified. The trial duration was shorter, and seat-on was earlier in the forward leaning strategy (trial duration: exaggerated forward leaning: 1.36 s ± 0.32, forward leaning: 1.27 s ± 0.27; z = 1.99, p = 0.046; seat-contact: exaggerated forward leaning: 0.65 s ± 0.17, forward leaning: 0.54 s ± 0.10; z = 5.07, p < 0.001).

The trials identified as outliers are shown in Supplementary Fig. 1. Concerning our hypothesis, we can state that we did not find specific strategies for 2a, the different degrees of rollator assistance, but we did for 2b, the challenging floor condition.

#### Stand-to-sit movement strategies

**Figure 6:**
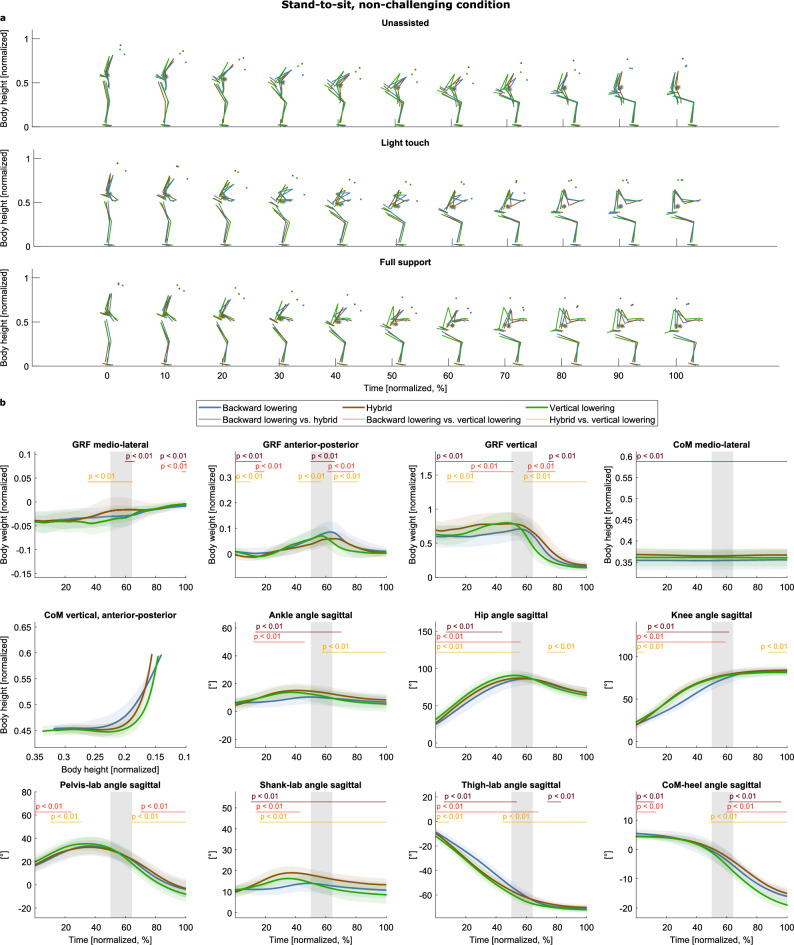
Strategies of the non-challenging stand-to-sit task. (**a**) The strategies are given in different colors (see legend) and separated into rows according to the support condition. The CoM is depicted as an asterisk (*). (**b**) Means and standard deviations of the variables, aggregated by cluster. The gray shaded area illustrates the range of seat-off (mean ± s.d.). The red lines and corresponding p-values indicate significant differences revealed by the post-hoc tests (p < 0.017).

Three strategies were found in the non-challenging condition (Fig. 6a, Supplementary Video “Stand-to-sit_non-challenging_condition.gif”): a “backward lowering” (blue), a “vertical lowering” (green), and a “hybrid” strategy (brown), inferred from visual inspection of the stick figures and supported by (1) the different CoM progressions and (2) the smaller hip, knee, and ankle angles of especially the backward-lowering strategy (Fig. 6b). Like with the sit-to-stand tasks, the three identified strategies appeared in each support condition.

Vertical lowering took less time, and seat-on was earlier than in the backward lowering and the hybrid strategy (trial duration: backward lowering: 1.47 s ± 0.29, hybrid: 1.49 s ± 0.26, vertical lowering: 1.33 s ± 0.24; χ^2^(3) = 17.41, p < 0.001; backward lowering vs. hybrid: z = −0.09, p = 0.925; backward lowering vs. vertical lowering: z = 2.60, p = 0.009; hybrid vs. vertical lowering: z = 4.24, p < 0.001; seat-on: backward lowering: 0.88 s ± 0.20, hybrid: 0.87 s ± 0.19, vertical lowering: 0.71 s ± 0.15; χ^2^(3) = 33.44, p < 0.001; backward lowering vs. hybrid: z = 0.14, p = 0.889; backward lowering vs. vertical lowering: z = 4.64, p < 0.001; hybrid vs. vertical lowering: z = 5.21, p < 0.001).

**Figure 7:**
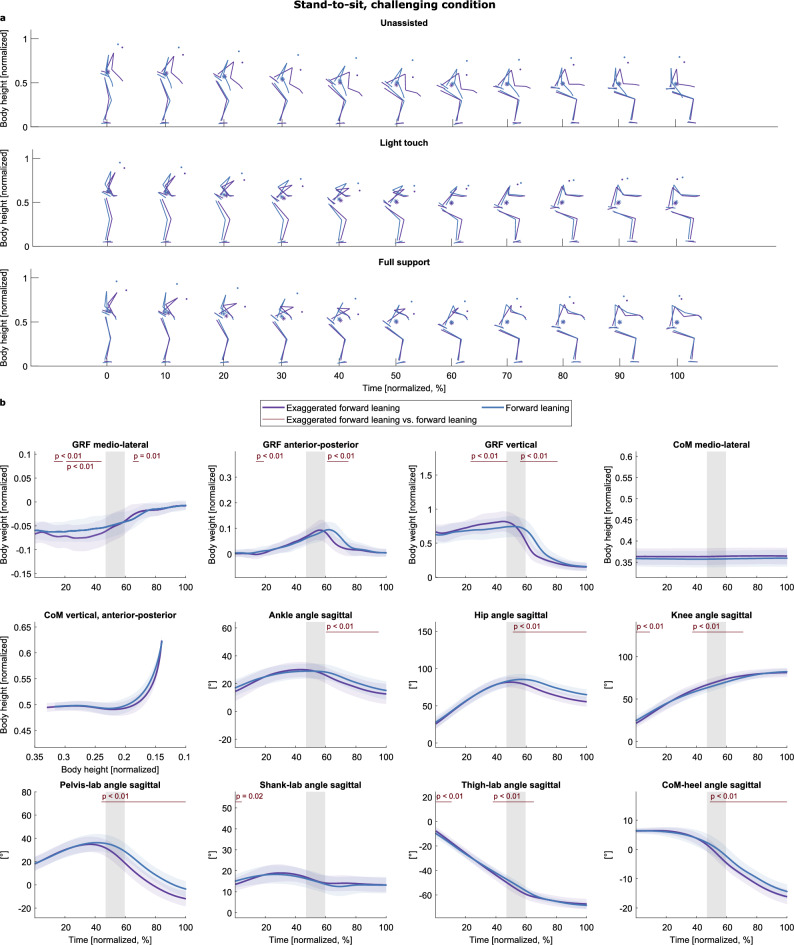
Strategies of the challenging stand-to-sit task. (**a**) The strategies are colored in different colors (see legend) and separated into rows according to the support condition. The CoM is depicted as an asterisk (*). (**b**) Means and standard deviations of the variables, aggregated by cluster. The gray shaded area illustrates the range of seat-off (mean ± s.d.). The red lines and corresponding p-values indicate significant differences revealed by the post-hoc tests (p < 0.017).

As with the sit-to-stand task, only two strategies were identified in the challenging condition (Fig. 7a, Supplementary Video “Stand-to-sit_non-challenging_condition.gif”): an “exaggerated forward leaning” (purple) and a “forward leaning” (blue) strategy. These strategies were inferred from visual inspection and underpinned by the smaller sagittal hip and ankle angles after seat-on in the exaggerated forward leaning strategy and the smaller CoM-heel angles after seat-on (indicating that the CoM is more anterior to the heel position than in the forward leaning strategy). As in the other task, no strategy was identified to comprise only trials with handle support. Exaggerated forward leaning took less time, and seat-contact was earlier than with forward leaning (duration: exaggerated forward leaning: 1.39 s ± 0.30, forward leaning: 1.47 s ± 0.29; z = − 2.01, p = 0.044; seat-on: exaggerated forward leaning: 0.70 s ± 0.15, forward leaning: 0.81 s ± 0.19; z = − 4.29, p < 0.001).

With respect to our hypotheses and in line with sit-to-stand, we can state that we did not find specific strategies for 2a, the different degrees of rollator assistance, but we did for 2b, the challenging floor condition.

#### The use of the arms in unassisted sit-to-stand and stand-to-sit movements

**Figure 8:**
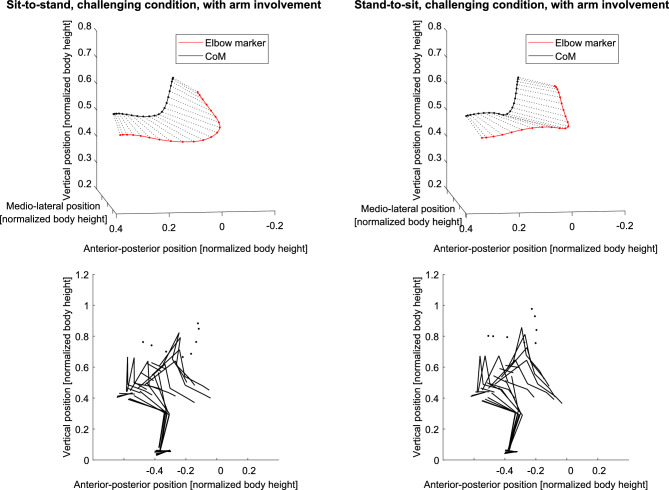
Example arm activity for two movements (left: sit-to-stand, right: stand-to-sit). Top: Spatial progression of the CoM and the lateral elbow marker. The black and red lines connecting the points illustrate the spatial progression. The distances between the points illustrate 5% of the total duration in each case. The cross-connections help assess whether the elbow and CoM moved at comparable speeds (as with nearly parallel cross-connections). Bottom: The whole-body movement is shown along with the plots above.

Arms were only used for two trials of the forward leaning and the hybrid strategy. In the challenging condition, 20 trials included arm movement, 15 of which belonged to the exaggerated forward leaning strategy (Fig. 8). In stand-to-sit, arms were not involved in the backward lowering, but five were involved in the vertical lowering and two in the hybrid strategy. When challenged, arms were involved in four trials in the exaggerated forward and two trials in the forward leaning strategy (Fig. 8). As inferred from the parallel lines in Fig. 8, arms were moved at a similar speed to the CoM, i.e., not swung.

## Discussion

Rollators are often prescribed to assist older individuals in their daily life, but paradoxically have been found to increase fall risk. Standing up and sitting down are often executed, yet are complex and demanding movements for older individuals. To improve this movement for older individuals, this study with young, non-disabled individuals identified if and how rollators affect movement strategies. We found that (1) most (14–17 of 20, task-dependent) participants switched their strategies with rollator assistance, (2a) no support-specific strategies were revealed, but (2b) new strategies emerged when participants’ balance was challenged.

### Participants switch their strategies with full support, especially when challenged

Participants who stood up with the forward leaning strategy in the unassisted condition did not show a common pattern of which strategy they switched to when having the handle support: some kept leaning forward, and others switched to a vertical rise or hybrid strategy. However, those who used the vertical rise or hybrid strategy unassisted never used the forward leaning strategy with handle support. When their balance was challenged, most participants (16 of 20) switched from the exaggerated forward to the forward leaning strategy with full support. During the light touch trials, one-third used the exaggerated and two-thirds the forward leaning strategy, i.e., presumably the somatosensory input is not the entire reason for changing the strategy. In stand-to-sit, almost all participants who used the backward lowering strategy in the unassisted condition switched their strategy in the full support condition, but not all switched in the light touch condition. When challenged, almost every trial with full support belonged to the forward leaning strategy, i.e., participants who used the exaggerated forward leaning strategy in the unassisted and/or light touch condition switched with full support.

Taken together, full handle support appears to influence strategy selection, especially when the participants were challenged, i.e., the demands on balance control were higher. Further, somatosensory input does not seem to determine the change in strategy under the studied task conditions. A change in the CoM position may be a potential triggering factor for strategy selection. Having the arms on the handles may lead to an anterior shift of the CoM, though rather small, due to the relatively small mass of the arms. This is supported by  Jeyasurya et al.^102^, who did not find significant differences in the CoM position when they compared an arm-assist and bar-assist position to the unassisted condition (arm crossed in front of the chest). Furthermore, the causal relationship between CoM-position and strategy selection is debatable. In fact, a recent study^103^ argues that the CoM represents the whole body well but questions if the CoM position/movement is optimized regarding a task goal. Hence, it remains an open question if the CoM position may trigger the strategy selection or if anything else triggers the strategy selection resulting in a shifted CoM position. According to a prevailing theory in motor control, the optimal control theory^104^, movement strategy selection is based on a cost function that is minimized about the movement goal (e.g., to stand up or sit down). Therefore, a unique control policy is implemented to transform the state estimate (i.e., the internal body representation and task-relevant variables) into motor commands^105^. The multi-dimensional state estimate could be considered a multi-dimensional input weighted to yield the selected movement strategy. A shift in the weighting, e.g., toward a higher importance of stability in the challenging condition, may explain a change toward a different, then optimal, movement strategy. A previous study^106^ proposed that this could explain age-related differences in movement strategies.

### Sit-to-stand was achieved with three different strategies

Our data-driven approach revealed three movement strategies (forward leaning, hybrid, and vertical rise). While these three have often been described in unassisted standing up^33,37,39,40,44,107^, we can now add that rollator assistance does not lead to the emergence of new strategies. In line with the literature, we found that participants using the forward leaning strategy showed higher upper body flexion^40,44^ and stood up faster^33^. In Hughes et al.’s study^33^, participants were older than in our study. Thus, we can now conclude that the forward leaning strategy is faster regardless of the individual’s age. Scarborough et al.^43^ found that knee moment is smaller with the vertical rise strategy. However, more muscle activity around the knee is needed due to lack of generated upper body momentum. Possibly, rollator use reduces the demand on muscle activity around the knee, as has been shown for walking^108^. To recommend the best strategies, which are safe and with the least demand on knee moment, for older individuals to stand up with a rollator, future studies may investigate muscle activity and joint load in the vertical rise and hybrid strategies with rollator support.

Interestingly, no different strategies were identified for the light touch and full support compared to the unassisted condition. Thus, receiving sensory input and/or weight support to stabilize oneself did not change the movement, even when the balance was challenged. Possibly, the identified strategies are optimal solutions to achieve the task goal, i.e., to stand up, so no new strategies need to emerge. De Rugy et al.^109^ proposed that habitual movement strategies might be retained when the task goal can be achieved, even if conditions change. This could have been the case with our young participants.

### When the balance was challenged, participants only used two different strategies to stand up

Participants used an exaggerated forward or forward leaning strategy when their balance was challenged. They did not use a third, vertical rise strategy as they did under non-challenging conditions. An exaggerated forward leaning strategy, based on increased trunk flexion, has been found in older individuals^110^ and in those with pathologies, when stability and safety seem to be of priority, thus often called “stabilization strategy” or “exaggerated trunk flexion strategy”^37,40,43,111,112^. Here, participants moved their CoM over the base of support (“stable position”) at the earliest time point by an exaggerated trunk flexion and then raised the CoM vertically. This behavior hints at why we found this strategy. The balance pads under the participants’ feet contained air, which was re-distributed when the pressure point from above changed. It seems plausible that the transitioning is easier when the CoM is centered above the balance pad at seat-off (CoM-heel angle ≈ 0°, see Fig. 5b).

Furthermore, the constantly changing demands due to the pressure distribution in the pads possibly required constant adjustment of the motor commands, as has been shown in studies on postural control^113^. The higher number of principal components extracted for all GRF directions (Table 1) reflects this by indicating a more complex time series structure^79^. Thus, the emergence of the exaggerated forward leaning strategy could result in participants wanting to increase their stability and safety to better cope with the increased balance challenge.

When using handles, the choice in favor of the forward leaning strategy could hint that participants cope with the challenging floor condition by relying more on the rollator handles^114^: e.g., to lessen the pressure on the pads and so the difficulty standing on them, or to interact with the handles correcting unforeseen instabilities during the transition. This issue can be addressed in more detail in future studies, e.g., with instrumented handles. Possibly, as the hip angle is larger in the forward leaning strategy, this strategy is more suitable for individuals with limited range of motion e.g., due to issues like lower-back-pain^115^.

That the participants showed two instead of three strategies to successfully perform the sit-to-stand task suggests that young individuals are flexible concerning task demands^37^. The vertical rise strategy is possibly not a good solution for this new task demand: here, the CoM-heel angle does not reach 0° until after seat-off (see Fig. 4b). This could explain why the participants did not use it. Furthermore, we suggest balance pads can be a way to provoke young individuals to use sit-to-stand strategies observed with older individuals or those with pathologies.

### Stand-to-sit was achieved with three different strategies

Our data-based approach identified three strategies for sitting down: backward lowering, hybrid, and vertical lowering. To the best of our knowledge, no previous study explicitly identified strategies in the stand-to-sit movement or performed detailed biomechanical measurements on this motion. Only Dubost et al.^35^ observed two general patterns comparable to our study's backward and vertical lowering strategies, although they did not explicitly name them. The main difference between the two patterns they found was the more vertical orientation of the trunk (like in our vertical lowering strategy) in the one predominantly used by their older participants. Their young participants flexed their trunks more (like in our backward lowering strategy).

In contrast to^35^, in our study the young participants only used the backward lowering strategy to sit down without rollator support in two of 60 trials. However, unlike in our study, participants had to cross their arms in front of their chests, which makes movement less natural, as supported by studies that evaluated the five-times-sit-to-stand test^116,117^. Therefore, not imposing this restriction in our study may have fostered the use of the vertical lowering or hybrid strategy. In both strategies, seat-on occurred earlier than with the backward lowering strategy. Based on this measure, it is suggested that this is a safer strategy as the unstable transition phase is shorter^94^. However, this safety assumption needs to be investigated more thoroughly in future studies.

Unlike with the unassisted condition, we found the preferred strategies to sit down with full support to be the backward lowering and hybrid strategies. Here, knee flexion happens more slowly but more steadily. Possibly, this reduces the knee load and muscle activity demand, and handle support helps carrying out this strategy. However, future studies may investigate the three strategies in every condition by imposing these on participants. An examination of muscle activity, interaction forces, and joint moments may then improve understanding of which strategies are optimal for older rollator users.

### When the balance was challenged, participants only used two different strategies to sit down

With challenged balance, participants used either a forward leaning or an exaggerated forward leaning strategy to sit down. Like with the sit-to-stand task, participants demonstrated only two instead of three strategies. The backward lowering strategy was not used. With the exaggerated and forward leaning strategies, the CoM is first lowered vertically, presumably to keep the pressure ratio in the balance pads as constant as possible. The rearward movement occurs later (CoM-heel angle ≈ 0°, not until seat-off, see Fig. 7b). Probably, shifting the CoM posteriorly earlier, like in backward lowering (CoM-heel angle ≈ 0° slightly earlier than seat-off, see Fig. 6b), would lead to the balance pads filling increasingly in the front, resulting in more air in the anterior area of the balance pads and so the body moving posteriorly. Like with standing up, the higher number of principal components in the GRF variables compared to the non-challenging condition indicates a more complex time series and, thus, constant adjustments of motor commands^79^. This more complex pattern possibly triggered the strategy selection toward a “safer” strategy. Future studies could examine sitting down with older participants and/or those with pathologies. It seems plausible that the exaggerated forward leaning strategy could arise there as a counterpart to the "stabilization strategy" of the sit-to-stand movement.

### Arm usage in unassisted sit-to-stand and stand-to-sit does not lead to new strategies

For standardization reasons, arm movement is often restricted, e.g., the arms are to be crossed in front of the chest^37,40^. This, however, hinders the participants from performing the transitions naturally, as indicated by validation studies for the five-times-sit-to-stand test^116,117^. Furthermore, individuals could possibly keep to their strategy when using the arms to compensate for difficulties^40,106^. We only instructed our participants to let their arms hang laterally in the sitting position in the unassisted condition. Trials with arm usage were not identified as outliers, nor did they lead to a new strategy cluster. This is in line with Millington et al.’s^118^ study, in which angles and moments of the trunk, pelvis, and knee remained similar regardless of participants moving to flex their shoulders or elbows in unassisted standing up. Further, Carr and Gentile^47^ found that trunk flexion or peak horizontal and vertical momentum of the CoM did not differ between restricted and flexible arm movement conditions. Hence, arm use does not lead to a new movement strategy. Standing up from a chair at knee height and with “natural” speed may not create difficult task demands for young individuals. Therefore, arm use may not be necessary for them and therefore not done. This is in contrast to the challenging condition, where arms were involved in 23 of 60 unassisted trials. As participants moved their arms at a comparable speed to the CoM (Fig. 8), rather than using the swing to create extensive, additional momentum as is often done to increase height in jumps^119,120^, they probably used their arms to shift the CoM anteriorly^121^. This possibly helps to hold the CoM as long as possible over the pads (CoM-heel angle ≈ 0°) in the exaggerated forward leaning strategy. In stand-to-sit, arms were only used in seven (non-challenging) and six (challenging) of 60 unassisted trials. Here, similar to the sit-to-stand movement in the challenging condition, participants did not extensively swing their arms but seemed to hold them anteriorly, possibly also to hold the CoM in the same horizontal position.

### Limitations

Several potential shortcomings need to be considered. First, our simulator device is heavier than a commercially available rollator and can neither dip nor move horizontally, even if the handles are pulled or pushed on heavily. Thus, the outlier “pulling strategy” (Supplementary Material), where one participant extensively pulled on the handles to stand up, would not be possible with a real-world rollator. Nevertheless, pulling extensively on rails that are fixed to propel the body upward has been observed as a strategy in older individuals to stand up and sit down^122,123^ and should therefore be considered in future studies examining older individuals. Secondly, although not uncommon in other biomechanical studies^40^, for standardization purposes, we restricted the foot placement to be parallel and underneath the knees, which hindered the participants from pulling their feet backward to stand up, as it is sometimes observed^36^. Thirdly, we applied PCA and k-means clustering. It cannot be excluded that other methods would have led to a different constellation. However, these methods are well-established and frequently used in biomechanics^80,84,87^, and the allocation of the trials into the identified strategy clusters is plausible and robust. Fourthly, to the best of our knowledge, there are no criteria based on which trials can be assigned a movement strategy in rollator-assisted sit-to-stand and stand-to-sit movements. Thus, there was no prior knowledge regarding the number and composition of the movement strategy clusters, precluding an a priori power analysis. Hence, we used a common unsupervised, data-driven approach to identify movement strategies. With unsupervised algorithms, it is unforeseeable how the data are clustered. Consequently, the number of participants and trials were selected not based on an a priori power analysis but on comparable studies^25,38,66,67^. Fifthly, statistics between the strategies were calculated as if the trials between the clusters were independent and single observations. Therefore, these need to be treated with caution. However, statistics were only applied here to give an impression of where the time series tend to differ and to help qualitatively describe the strategies. Lastly, we investigated strategies in young people. Even though we used balance pads to challenge them, this probably limits the validity of the results concerning individuals who are older or physically limited and dependent on a rollator.

## Conclusion

This study found that young individuals switch their strategies to standing up and sitting down with rollator handle support and when their balance is challenged. Our data-driven approach revealed three strategies for sit-to-stand. The strategies found are in line with the literature on unassisted standing up. They have now been shown to hold with rollator support. Our challenging floor condition approach in using balance pads under the participants’ feet provoked two strategies, one of which was previously found for older individuals and those with pathologies. We suggest balance pads can be used in future studies as a way to provoke young individuals to use sit-to-stand strategies observed with older individuals or those with pathologies. For the first time, strategies for stand-to-sit have been described based on biomechanical data. Participants used three sit-down strategies, reduced to two in the challenging balance condition. Like with sit-to-stand, rollator support and challenging balance conditions greatly influence strategy selection. In our study, the strategies have been described and discussed predominantly based on kinematics. Future studies may investigate joint loading and muscle activity to assess which strategies can eventually be recommended to fall-prone individuals for safe and efficient rollator usage.

### Supplementary Information

Supplementary Information 1.

Supplementary Information 2.

Supplementary Information 3.

Supplementary Information 4.

Supplementary Figure 1.

## Data Availability

Correspondence and requests for materials should be addressed to the corresponding author Michael Herzog (michael.herzog@kit.edu).
