# Supplementary figures and images for: Rollator usage lets young individuals switch movement strategies in sit-to-stand and stand-to-sit tasks
Source: Sci Rep. 2023 Oct 6;13:16901. doi: 10.1038/s41598-023-43401-6 (PMC10558536; doi:10.1038/s41598-023-43401-6)

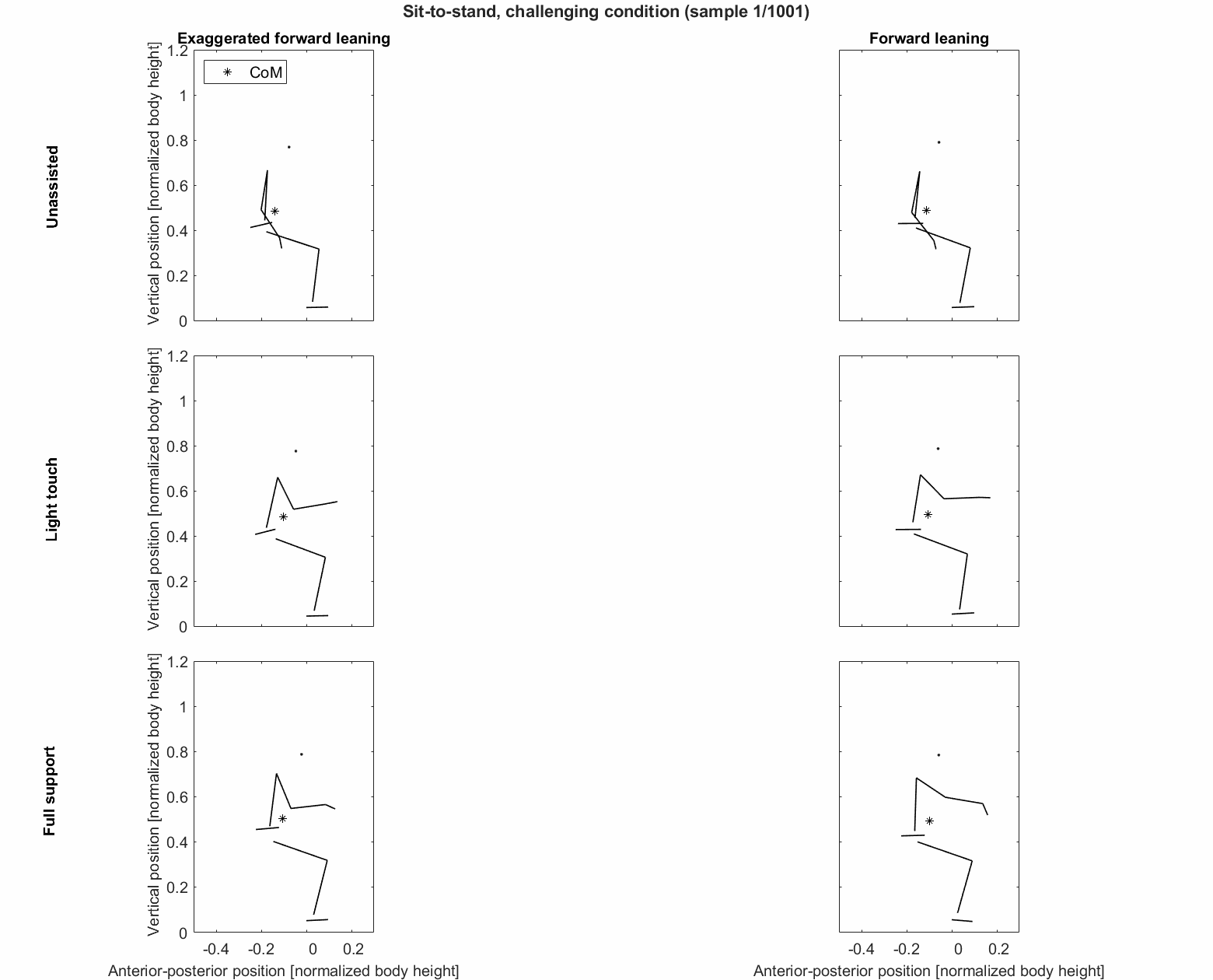

Supplement: Supplementary file 1 — Supplementary Information 1. [file 41598_2023_43401_MOESM1_ESM.gif]

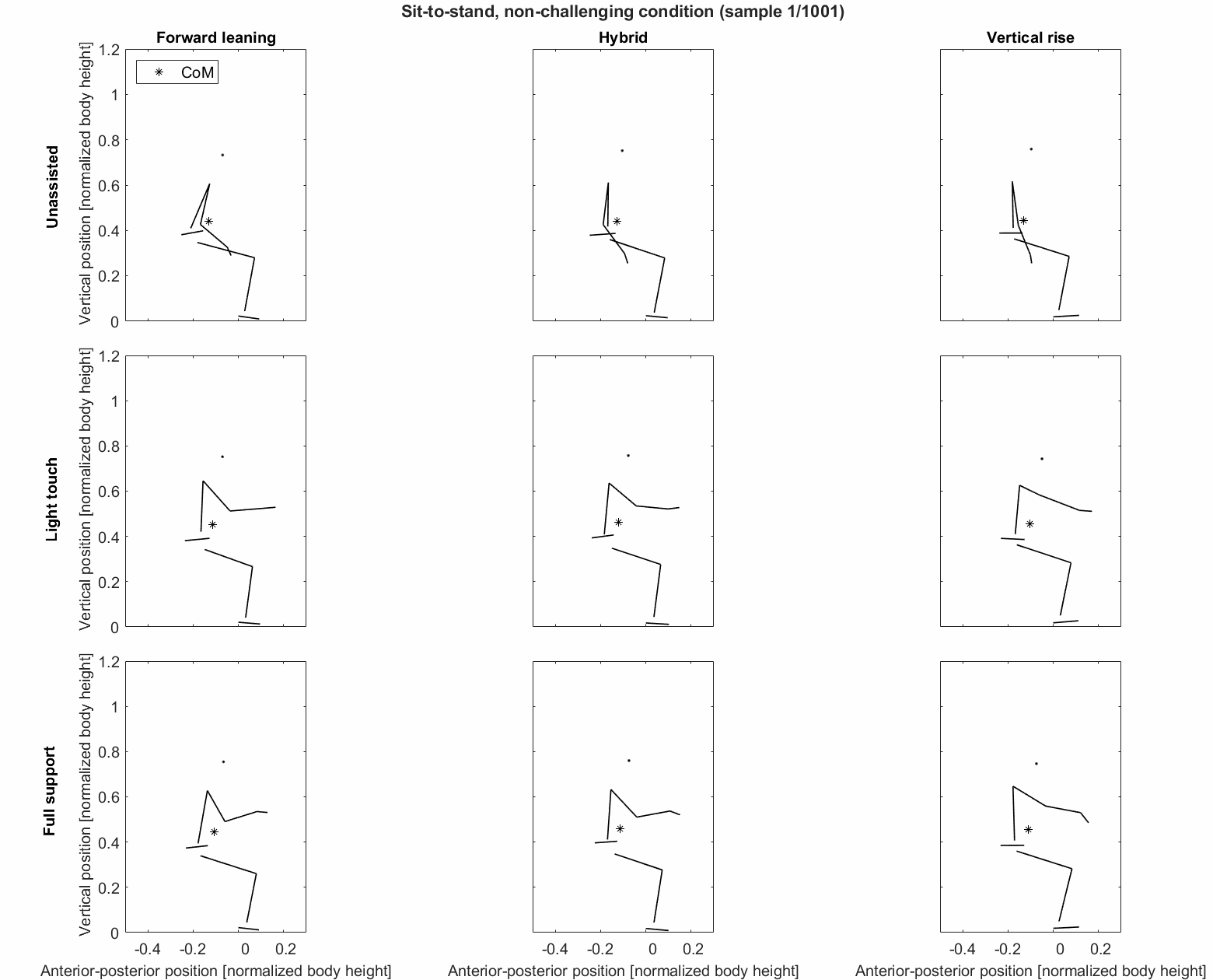

Supplement: Supplementary file 2 — Supplementary Information 2. [file 41598_2023_43401_MOESM2_ESM.gif]

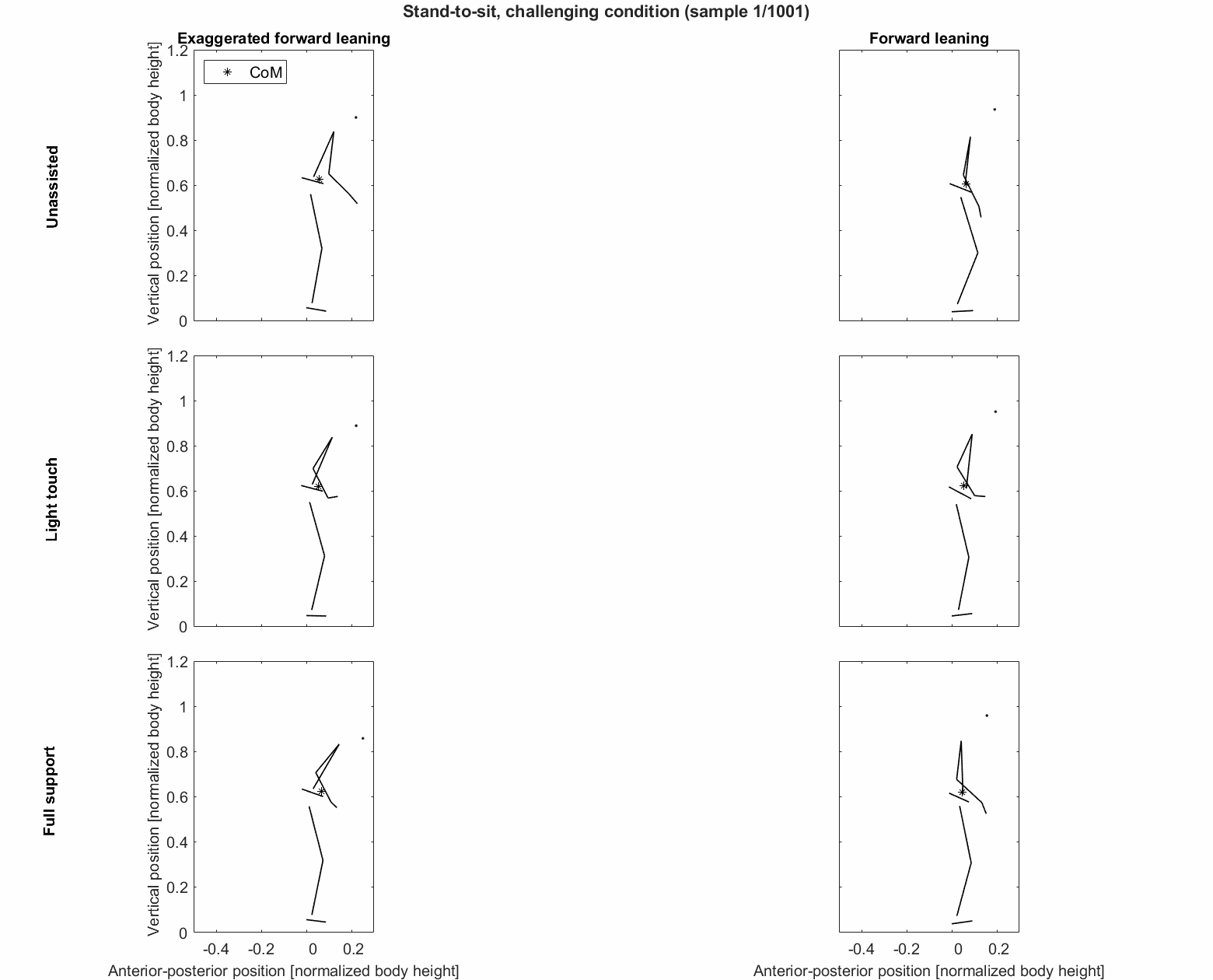

Supplement: Supplementary file 3 — Supplementary Information 3. [file 41598_2023_43401_MOESM3_ESM.gif]

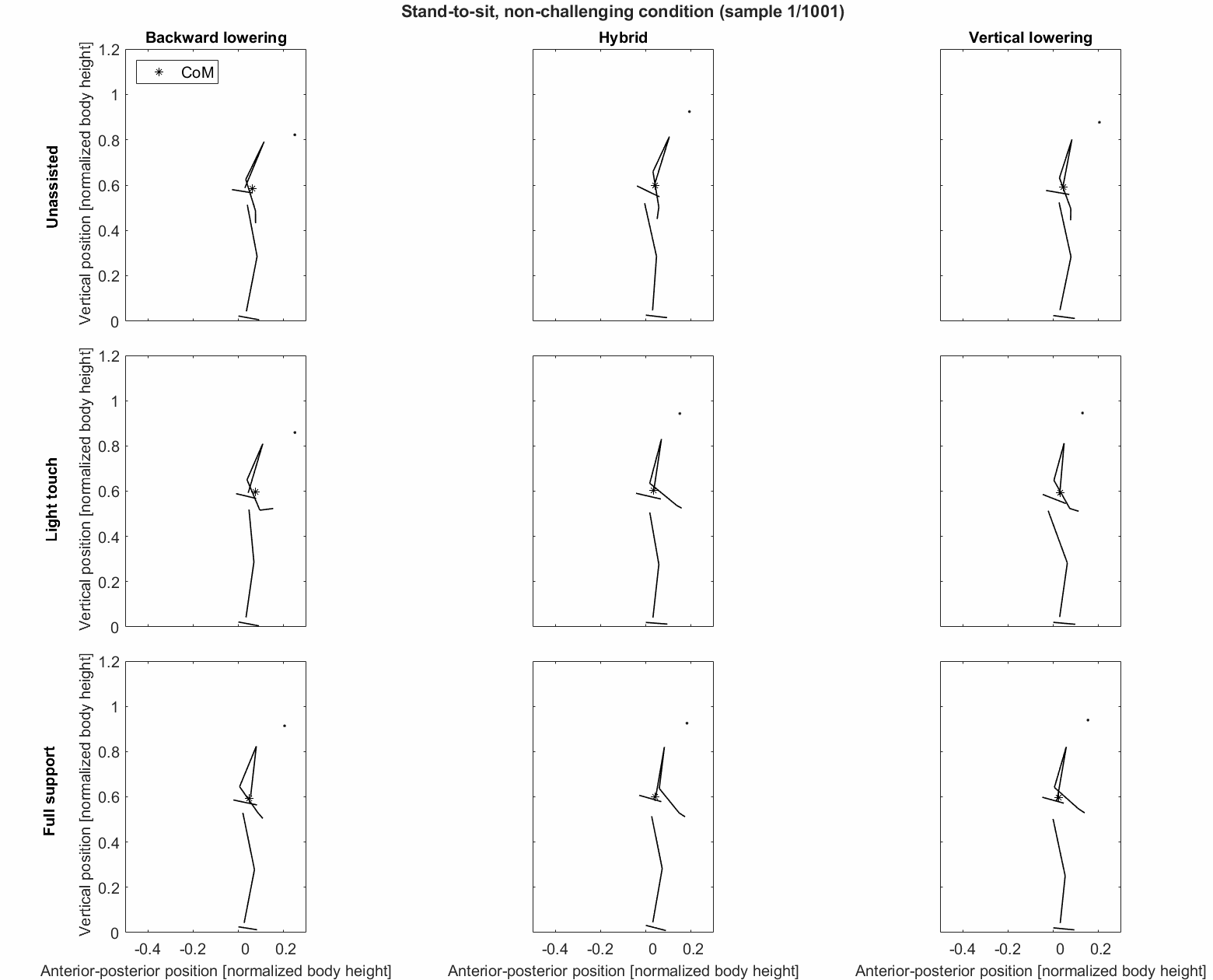

Supplement: Supplementary file 4 — Supplementary Information 4. [file 41598_2023_43401_MOESM4_ESM.gif]
