# Supplementary material for: Rollator usage lets young individuals switch movement strategies in sit-to-stand and stand-to-sit tasks
Source: Sci Rep. 2023 Oct 6;13:16901. doi: 10.1038/s41598-023-43401-6 (PMC10558536; doi:10.1038/s41598-023-43401-6)
Supplement: Supplementary file 5 — Supplementary Figure 1. [file 41598_2023_43401_MOESM5_ESM.pdf]

Supplementary Material

Rollator Usage Lets Young Individuals Switch Movement Strategies in Sit-to-Stand and Stand-to-Sit Tasks

Michael Herzog[1,2*], Frieder C. Krafft[2,3,4], Bernd J. Stetter[1,5], Andrea d'Avella[6,7], Lizeth H. Sloot[2,3], Thorsten Stein[1,2]

[1] BioMotion Center, Institute of Sports and Sports Science, Karlsruhe Institute of Technology (KIT), Karlsruhe, Germany
[2] HEiKA – Heidelberg Karlsruhe Strategic Partnership, Heidelberg University, Karlsruhe Institute of Technology (KIT), Germany
[3] Optimization, Robotics, and Biomechanics (ORB), Institute of Technical Engineering (ZITI), Heidelberg University, Germany
[4] Center of Prevention, Diagnostic and Performance, Center of Orthopaedics Hohenlohe, Künzelsau, Germany
[5] Sports Orthopedics, Institute of Sports and Sports Science, Karlsruhe Institute of Technology (KIT), Karlsruhe, Germany
[6] Laboratory of Neuromotor Physiology, IRCCS Fondazione Santa Lucia, Rome, Italy
[7] Department of Biomedical and Dental Sciences and Morphofunctional Imaging, Università di Messina, Messina, Italy

* Correspondence:
Michael Herzog
Karlsruhe Institute of Technology
BioMotion Center
Engler-Bunte Ring 15
76131 Karlsruhe, Germany
E-Mail: Michael.Herzog@kit.edu

Outlier trials in the sit-to-stand and stand-to-sit tasks

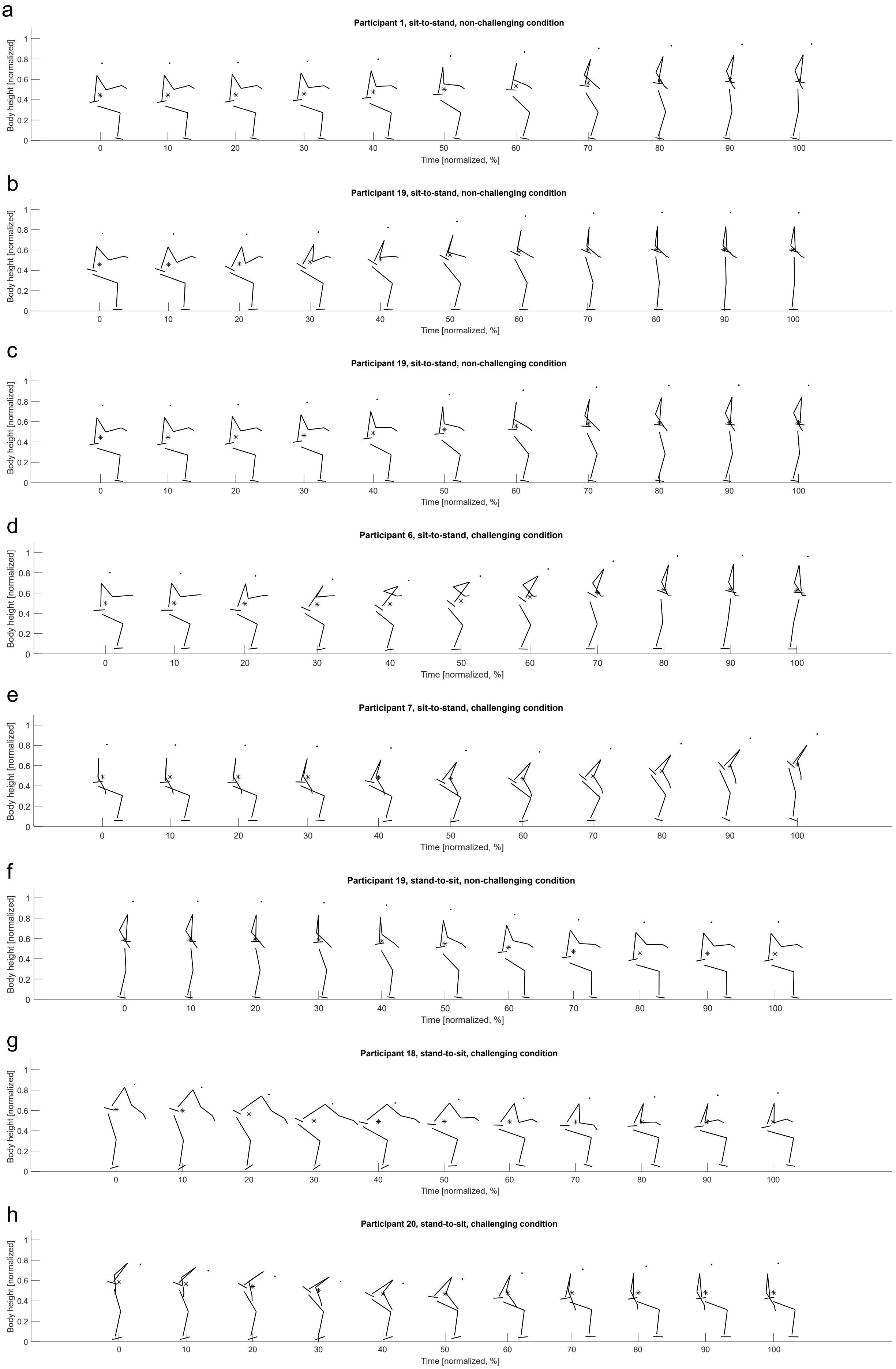

Supplementary Figure 1: Trials identified as outliers. These trials were first assigned to one of the strategies with k-means clustering but were later identified as outliers due to their large distance to the clusters' centroids. a-c: sit-to-stand, non-challenging, d-e: sit-to-stand, challenging, f: stand-to-sit, non-challenging, g-h: stand-to-sit, challenging condition. We call a-c “pulling strategy”, as this movement execution is only possible with extensive pulling on the handles.
